# Supplementary material for: Thiolated Chitosan Conjugated Liposomes for Oral Delivery of Selenium Nanoparticles
Source: Pharmaceutics. 2022 Apr 6;14(4):803. doi: 10.3390/pharmaceutics14040803 (PMC9032237; doi:10.3390/pharmaceutics14040803)
Supplement: Supplementary file 1 [file pharmaceutics-14-00803-s001.zip › pharmaceutics-1660009-supplementary.pdf]

Supplementary Material

Thiolated Chitosan Conjugated Liposomes for Oral Delivery of Selenium Nanoparticles

Atiđa Selmani [1,†], Elisabeth Seibert [2,†], Carolin Tetyczka [1,†], Doris Kühnelt [3], Ivan Vidakovic [2], Karin Kornmueller [2], Markus Absenger-Novak [4], Borna Radatović [5], Ivana Vinković Vrček [6], Gerd Leitinger [7], Eleonore Fröhlich [4], Andreas Bernkop-Schnürch [8], Eva Roblegg [1] and Ruth Prassl [2,*]

[1] Department of Pharmaceutical Technology and Biopharmacy, Institute of Pharmaceutical Sciences, University of Graz, 8010, Graz, Austria, atida.selmani@uni-graz.at, carolin.tetyczka@uni-graz.at, eva.roblegg@uni-graz.at

[2] Gottfried Schatz Research Center for Cell Signaling, Metabolism and Aging, Division of Biophysics, Medical University of Graz, 8010, Graz, Austria, elisabeth.seibert@uni-graz.at, ivan.vidakovic@medunigraz.at, karin.kornmueller@medunigraz.at, ruth.prassl@medunigraz.at

[3] Institute of Chemistry, Analytical Chemistry, NAWI Graz, University of Graz, 8010 Graz, Austria, doris.kuehnelt@uni-graz.at

[4] Center for Medical Research, Medical University of Graz, 8010, Graz, Austria, markus.absenger@medunigraz.at, eleonore.froehlich@klinikum-graz.at

[5] Center of Excellence for Advanced Materials and Sensing Devices, Institute of Physics, Bijenička 46, 10000 Zagreb, Croatiabradatovic@ifs.hr

[6] Institute for Medical Research and Occupational Health, Zagreb, Croatia, ivinkovic@imi.hr

[7] Gottfried Schatz Research Center for Cell Signaling, Metabolism and Aging, Division of Cell Biology, Histology and Embryology, Medical University of Graz, Graz 8010, Austria, gerd.leitinger@medunigraz.at

[8] Center for Chemistry and Biomedicine, Department of Pharmaceutical Technology, Institute of Pharmacy, University of Innsbruck, 6020, Innsbruck, Austria, andreas.bernkop@uibk.ac.at

*To whom correspondence should be addressed:

Ruth Prassl
Gottfried Schatz Research Center for Cell Signaling, Metabolism and Aging
Division of Biophysics
Medical University of Graz
Neue Stiftingtalstraße 6, 8010, Graz, Austria
Tel.: +43 316 385 71695
E-mail: ruth.prassl@medunigraz.at

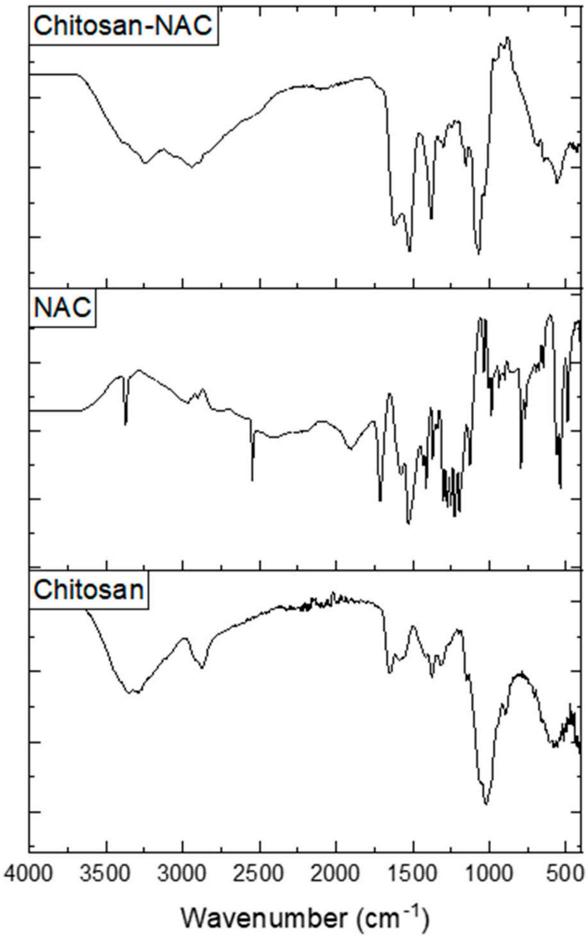

Figure S1. FT-IR spectra of chitosan, NAC and Cs-NAC after coupling. The absorption band in the FT-IR spectrum of chitosan was shifted from 1660 to 1625 cm^{-1} after modification. The appearance of a new amide peak at 1520 cm^{-1} (bending vibration of C-N-H, stretching vibration of C-N) and the shoulder in the range of 2590 and 2480 cm^{-1} belong to the thiol group in NAC.

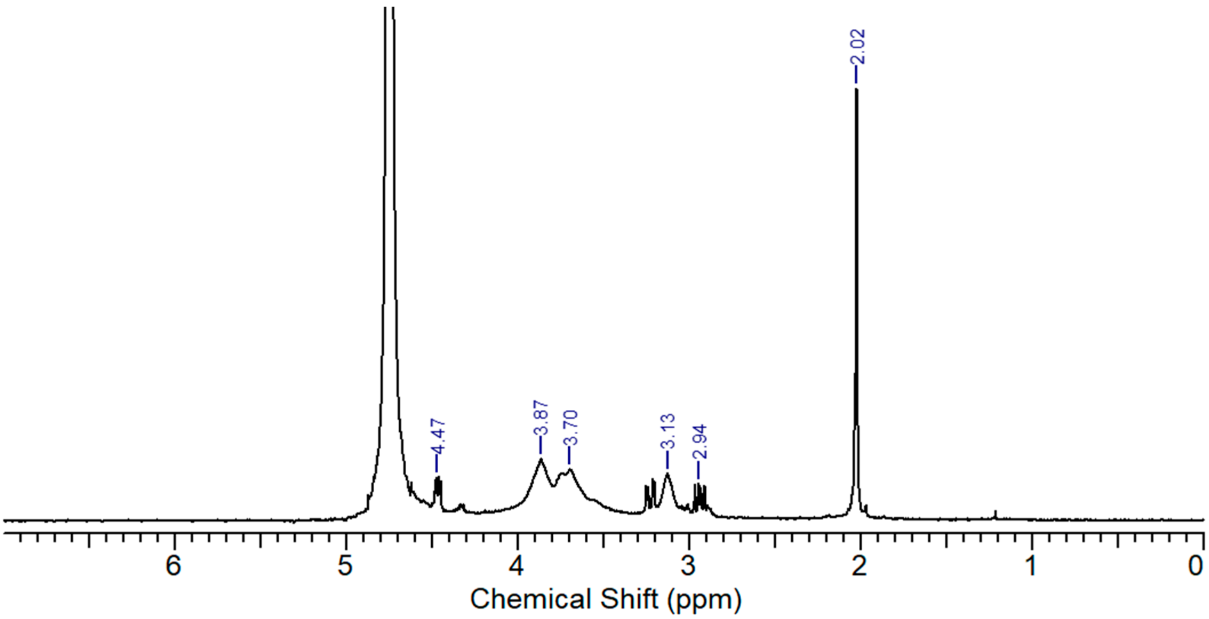

Figure S2. ^{1}H NMR spectrum of Cs-NAC. The chemical shift observed at 2.94 ppm belongs to the methylene protons next to thiol moiety, while the one at 4.47 ppm to the methine protons in NAC. At 2.02 ppm, the peak of the acetyl protons appeared with high intensity, as an indication of the excess of acetyl groups, compared to native chitosan.

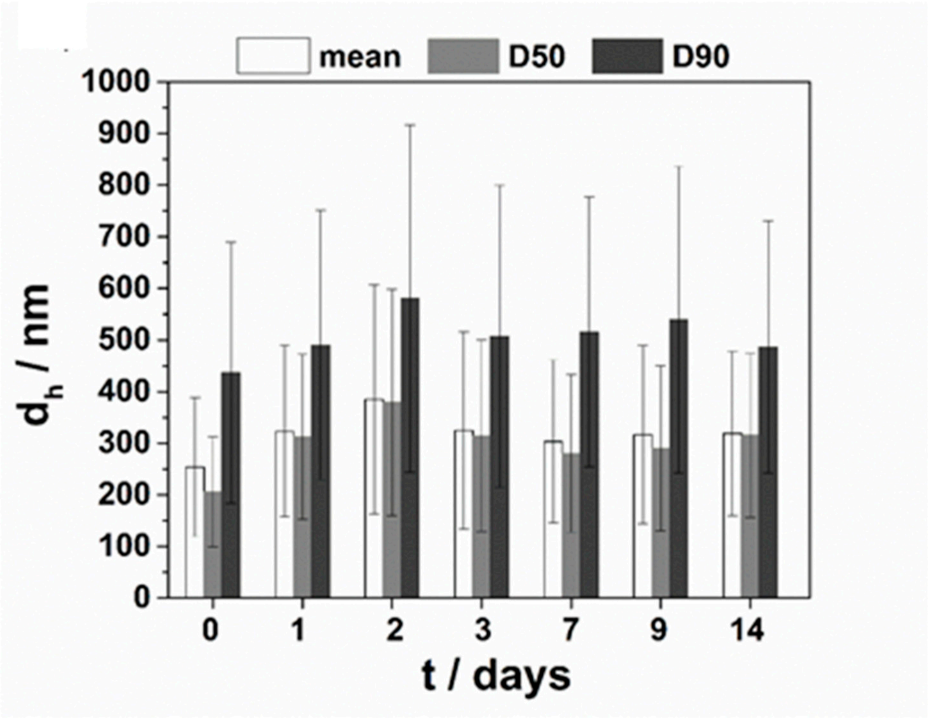

Figure S3. Particle size determination of thiolated Lip-SeNPs using NTA. Particle size distribution 50% (D50) and 90% (D90) values and the mean particle diameter are presented as mean values ± SD (n =3) for a storage time of 14 days at 4°C.

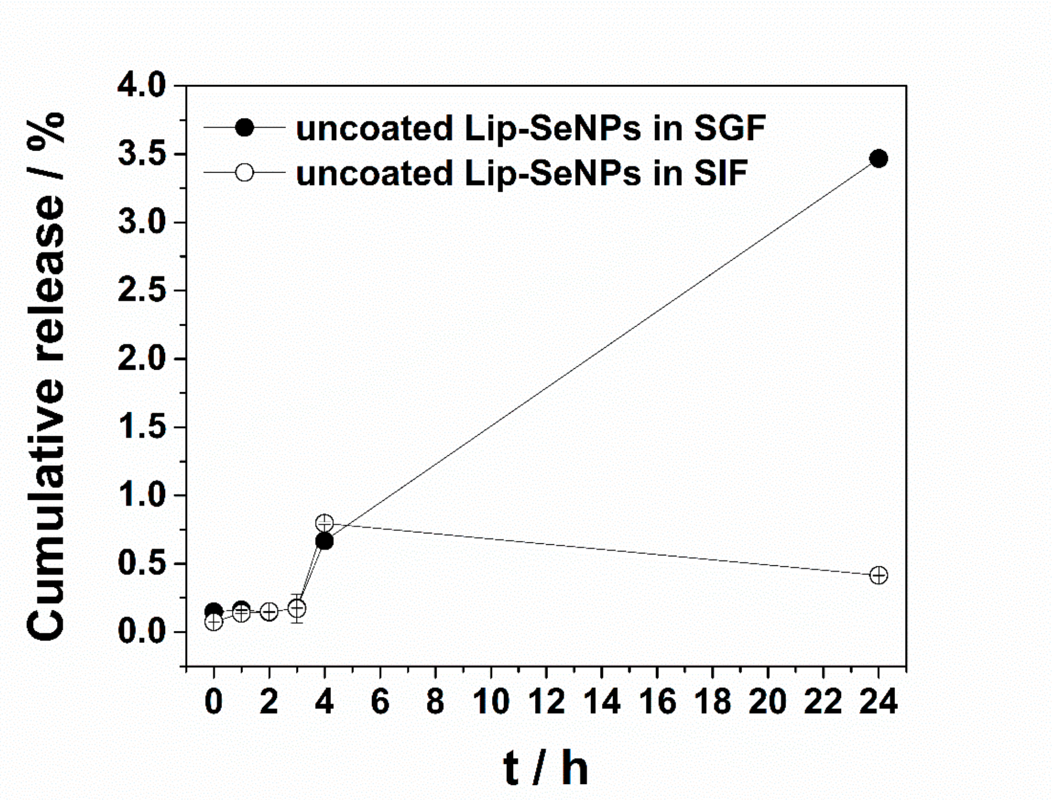

Figure S4. Cumulative release (%) of selenium from Lip-SeNPs at predetermined time points after incubation with simulated gastric fluid, SGF (filled symbols) and simulated intestinal fluid, SIF (open symbols) at 37 °C (n=2). The selenium concentration determined after membrane solubilisation with Triton-X was taken as 100% release.

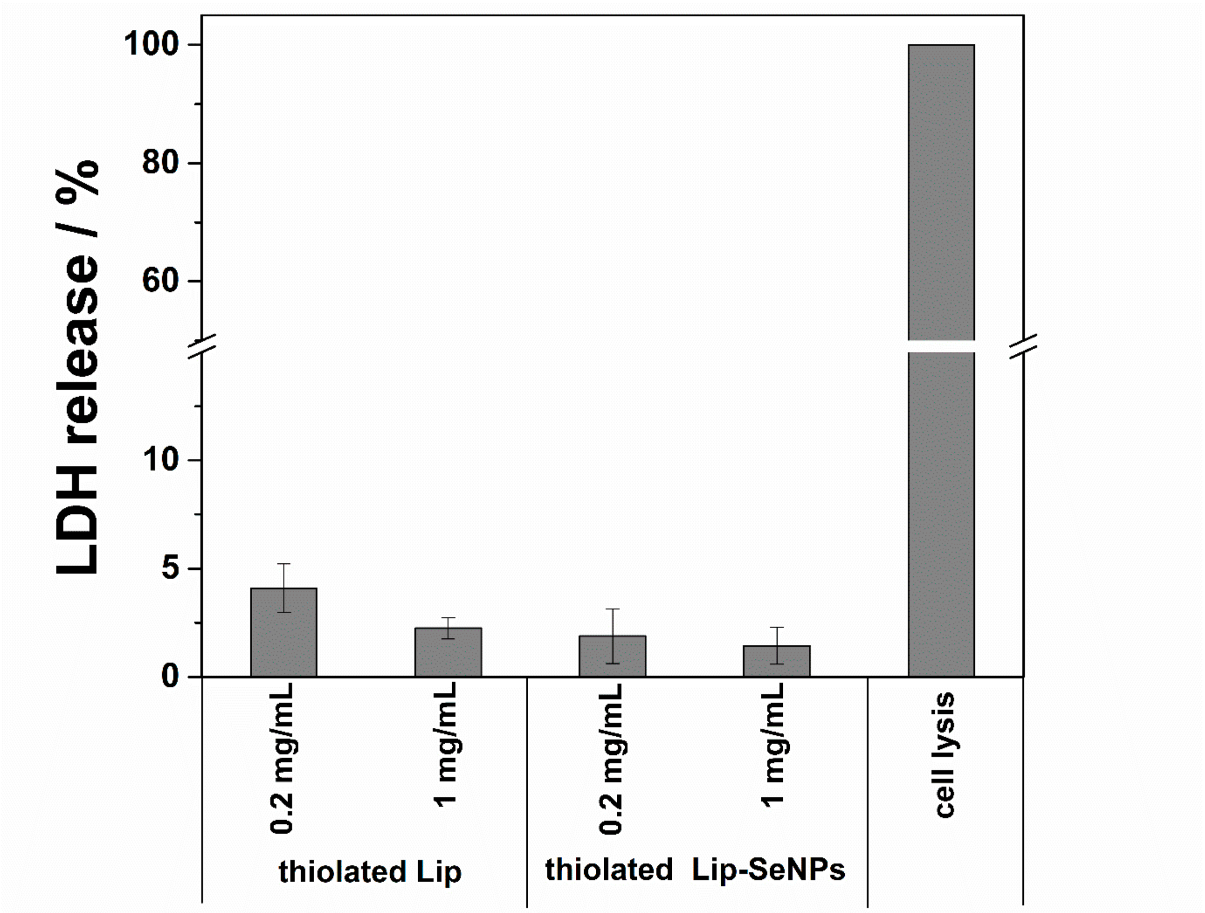

Figure S5. Cytotoxic effects of Lip-SeNPs and empty thiolated liposomes without SeNPs were determined on a co-culture model of Caco-2:HT29-MTX; 7:3, measuring the % LDH release. Untreated cells were used as control. Two sample concentrations with a lipid mass of 0.2 and 1 mg/mL corresponding to about 8 and 40 µg/mL of SeNPs, respectively, were incubated with the cells for 4h. The values are expressed as mean values ± SD (n=3).

Table S1. Fitting results for the SAXS pattern of uncoated and thiolated Lip-SeNPs.

	**Uncoated Lip-SeNPs**	**Thiolated Lip-SeNPs**
z_H	1.65 nm	1.60 nm
σ_H	0.3 nm	0.3 nm
ρ_R	1.09 (a.u.)	1.95 (a.u.)
σ_C	0.86 nm	0.58 nm
d_{HH}	3.3 nm	3.2 nm

Notes: The scattering curves were fitted with a bilayer form factor model. A Gaussian representation of the electron density profile was used. Abbreviations: z_H is the center and σ_H the width of the head group Gaussian; σ_C, the width of the hydrocarbon chain Gaussian; and ρ_R, the ratio of the methyl-terminus electron density amplitude to the headgroup amplitude ($\rho_R = \rho_C/\rho_H$) in arbitrary units (a.u.). The headgroup-to-headgroup distance (d_{HH}) is defined as 2_{zH}.
